# Aberrant expression of PI3K/AKT signaling is involved in apoptosis resistance of hepatocellular carcinoma

**DOI:** 10.1515/biol-2021-0101

**Published:** 2021-09-27

**Authors:** Zhuangqiang Wang, Xiaopeng Cui, Gaopeng Hao, Jiefeng He

**Affiliations:** Department of Hepatobiliary Surgery, Shanxi Bethune Hospital, Shanxi Academy of Medical Sciences, Tongji Shanxi Hospital, Third Hospital of Shanxi Medical University, No. 99 Longcheng Street, Taiyuan 030032, Shanxi, China; Department of General Surgery, Shanxi Provincial People’s Hospital, Taiyuan 030032, Shanxi, China

**Keywords:** caspases, cell death, liver cancer, PI3K/AKT signaling, PTEN

## Abstract

Phosphatidylinositol 3-kinase (PI3K)/AKT signaling is a crucial pathway for cell survival and proliferation, which are regulated by several growth factors and activated receptors. Upregulated PI3K/AKT signaling molecules were reported in several cancers and they are associated with altered cellular functions, leading to oncogenesis. Here, we have examined the implications of elevated PI3K/AKT expression in the apoptosis resistance of human hepatocellular carcinoma (HCC) Huh7 cells. We showed that PI3K/AKT signaling is significantly upregulated in Huh7 cells by quantitative polymerase chain reaction and protein expression analysis. Also, perversely upregulated PI3K/AKT signaling Huh7 cells are highly resistant to treatment with chemotherapy drugs (docetaxel and sorafenib) and acquired apoptosis resistance through downregulation of tumor suppressor protein PTEN (phosphatase and tensin homolog deleted on chromosome ten). Hence, we have investigated the effect of PTEN overexpression on apoptosis induction in Huh7 cells. We showed that PTEN overexpressed Huh7 cells became more sensitive toward the aforesaid drugs and induced apoptotic cell death due to intracellular reactive oxygen species (ROS) generation. Concurrently, the overexpression of PTEN leads to the activation of mitochondria facilitated intrinsic apoptosis, evidenced by upregulated cytochrome C, caspase 3, and caspase 9. Collectively, our data suggest that the aberrant expression of PI3K/AKT signaling contributes to apoptosis resistance in HCC.

## 1 Introduction

Liver cancer or hepatocellular carcinoma (HCC) belongs to the most prevalent malignancies and has become the third underlying cause for cancer-allied deaths worldwide, accounting for more than 700k deaths per year [1,2]. Treatment strategies include surgical resection and chemotherapy. However, surgical resection endures the first line of choice for patients with smaller tumors with conserved liver function without vascular invasion [3]. Besides advancement in HCC treatment regimens, the outcome of the patient’s survival rate remains poor due to tumor relapse and intra- or extrahepatic metastasis, which occurs within 2 years of surgery [4]. Several reports have demonstrated that deregulation of signaling pathways, transcriptional factors, growth factors, genes, and proteins play a critical role in HCC oncogenesis [5,6]. The key signaling pathways involved in HCC tumorigenesis include WNT/β catenin, angiogenic signaling, hepatocyte growth factor/c-MET, ERK, PI3K/AKT, and mTOR [7,8,9,10,11].

The phosphatidylinositol 3-kinase (PI3K)/AKT signaling pathway particularly performs essential cellular functions, like cell growth, proliferation, and survival. Hence they are termed as “cell survival pathways” [12]. Under normal conditions, PI3K activation leads to the production of phosphatidylinositol (3,4)-[PI(3,4)P2]/(3,4,5)-bisphosphate [PI(3,4,5)P3], which in turn stimulates Akt, a serine-threonine protein kinase B. Ultimately, the activated Akt acts as a secondary messenger and by phosphorylation it performs various cellular functions [13]. In this cycle, the PI3K/AKT signaling is negatively regulated by the phosphatase and tensin homolog (PTEN), a lipid phosphatase, which can remove phosphoric acid from PIP3 and is subjected to degradation [13,14].

Studies have shown that PI3K/AKT signaling tends to be aberrantly activated and overexpressed in HCC cells [15,16,17], which is crucial for epithelial–mesenchymal transition (EMT) and thus leads to HCC invasion and metastasis [17,18,19]. Activated PI3K/AKT signaling causes enhanced expression of matrix metalloproteinases and upregulate the snail transcriptional expression for EMT induction. Interestingly, several studies have reported that PI3K/AKT signaling molecules play a key role in sorafenib resistance in HCC cells [15,16,17]. However, the molecular mechanism or the factors involved in the apoptosis and chemoresistance of HCC cells are not well characterized so far. Therefore, elucidating such molecular mechanisms and causative factors involved in the resistance of chemotherapeutic drugs and apoptosis would improve the understanding of pathogenesis as well as the treatment modules of HCC. Considering these facts, this study was first designed to examine the expression pattern of PI3K/AKT signaling molecules in HCC Huh7 cells. Furthermore, we have investigated the factors involved in PI3K/AKT signaling-mediated apoptosis/chemoresistance in Huh7 cells.

## 2 Materials and methods

### 2.1 Cell culture

Human HCC Huh7 cells were purchased from the Cell Bank, Chinese Academy of Sciences (Shanghai, China). The normal (non-cancerous) human liver cells (hepatocytes) with normal functionalities were used as a control for all the experiments. Dulbecco’s modified Eagle’s medium (DMEM) supplemented with 10% FBS (DMEM and FBS from Gibco, Shanghai) and antibiotics were used for cell culturing at 37°C and 5% CO_2_.

### 2.2 Huh-7 origin and characteristics

The HuH-7 is an immortal, epithelial-like, well-differentiated hepatocyte cellular carcinoma cell line, which was established in 1982. It is originally derived from a 57-year-old Japanese male liver cancer patient. Huh-7 cells are adherent and able to grow in 2D cultures.

### 2.3 Reverse transcription polymerase chain reaction (RT-PCR) analysis

cDNA synthesis kit, RT-PCR/PCR kit, polymerases, and restriction enzymes were purchased from Ta Ka Ra Biotechnology, China. Total cellular RNA extraction was performed as per the protocol explained in the RNA extraction kit (Beijing Solar Biotechnology). The RT-PCR (Biorad) was performed as described previously [20]. The primers used for gene amplification were PI3K: F-AACACAGAAGACCAATACTC and R-TTCGCCATCTACCACTAC [21]; AKT: F-GTGGCAAGATGTGTATGAG and R-CTGGCTGAGTAGGAGAAC; PTEN: F-AAGGCACAAGAGGCCTAGATTTCT and R-ACTGAGGATTGCAAGTTCCGCCA [22]; and GAPDH: F-ATGTCGTGGAGT CTACTGGC, and R-TGACCTTGCCCACAGCCTTG [23]. The mRNA expression folds of PI3 and AKT were calculated using Ct values and normalized with GAPDH (house-keeping gene) as per the formula: 2−ΔCt [ΔCt = Ct target gene-Ct-GAPDH]. The quantification graph represents the average value from four individual experiments.

### 2.4 Transfection

Approximately 2 × 10^6^ cells/well were seeded in 6-well plates. After 24 h of culturing, cells were transfected with plasmids such as 809 pcDNA3-GFP-PTEN (Plasmid #10759) [24] or empty vector pCMV-PTEN (Plasmid #28298) by using 8 µL of the transfection reagent, Lipofectamine (Qiagen). After 24 h of incubation at 37°C in 5% CO_2_, cells were subsequently subjected to *in vitro* assays.

### 2.5 Flow cytometry analysis

After 48 h of transfection with PTEN overexpression Cassette, cells were fixed in ice-cold methanol (70%) and subjected to propidium iodide (PI) staining (PI staining kit from Sigma-Aldrich) at 37°C under dark conditions overnight. The next day, cells were subjected to flow cytometry analysis (BD Biosciences, USA). The rate of apoptosis was evaluated based on the mean fluorescent intensity of PI measured by flow cytometry. The mean intensity values are represented as a quantification graph. The values were taken from three individual experiments.

### 2.6 Intracellular ROS measurements

Cells were cultured for 48 h incubation, both non-transfected and GFP-PTEN transfected cells were washed by 1XPBS, and subsequently treated with 2′,7′-dichloro fluorescin diacetate (DCFH-DA) under dark conditions for 30 min. Subsequently, cells were subjected to flow cytometry to measure the mean intensity of fluorescence exhibited by DCFH-DA. The mean intensity values are represented as a quantification graph. The values were taken from three individual experiments.

### 2.7 Western blot analysis

Proteins were extracted and boiled in the 2× Biorad sample buffer, separated by SDS-polyacrylamide (10%) gel electrophoresis, transferred to polyvinylidene difluoride membranes, and subsequently blocked with 5% skim milk in 1× phosphate buffered saline/tween (PBST). Blots were probed with primary antibodies against Akt (1:1,000), PI3 (1:500), PTEN (1:1,500) (from Santa Cruz Biotechnology); caspase 3 (1:500), caspase 9 (1:500), Bcl-2 (1:2,000), cytochrome C (1:1,000), and GAPDH (1:5,000) from Beijing Zhongshan Biotechnology. Horse-radish peroxidase conjugated the secondary antibody (1:10,000) from the Cell Signaling Technology. The protein signal was visualized by 2 mL of ECL chemiluminescence (Biorad). The signal intensities of the protein band were quantified by FIJI image analysis software, and the signal values were normalized with the loading control GAPDH protein.

### 2.8 Apoptosis resistance assay

Cells were seeded in a range of 10^4^ cells/well and incubated overnight in 96-well plates. Then cells were treated with 10 μM of Docetaxel (Rhone-Poulenc Rorer Pharmaceuticals) and 10 μM of Sorafenib (Jinan Trio Pharmatech) and incubated for 48 h. Apoptosis resistance was evaluated exactly as described previously by using the formula: Cell resistance rate (%) = (experimental group OD_450_ value/control group OD_450_ value) × 100 [25].

### 2.9 Cell proliferation assay

Cell proliferation assay was performed in 6-well plate by seeding approximately 10^6^ cells/well. After 24 h of drug treatment (docetaxel and sorafenib) or transfected cells (PTEN overexpression), the rate of cell proliferation was measured by treating cells with 10 μL of cell counting kit-8 solution for 2–3 h. Then the growth rate was measured at 450 nm and the values (from the three independent experiments) obtained were represented in the quantification graph.

### 2.10 Statistical analysis

The values denoted in the graphs were mean ± SD. The Student *t* test was performed for statistical analysis and the one-way analysis of variance was used to compare the two groups. When the *P*-value was **P < 0.01 and *P < 0.05, they were considered as statistically significant.

## 3 Results

### 3.1 Activation of PI3K/AKT signaling pathway is associated with apoptosis resistance in Huh7 cells

**Figure 1:**
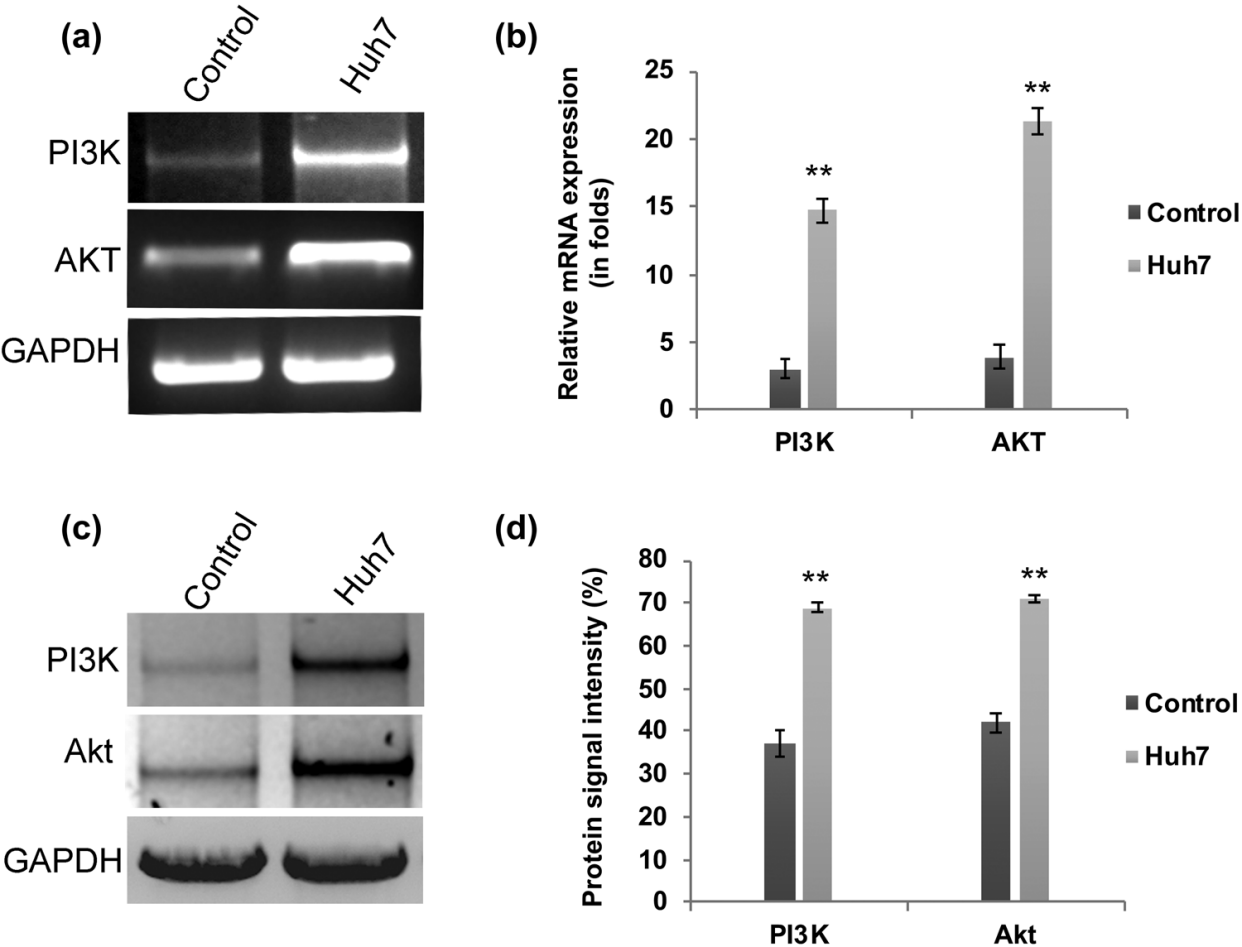
Activation of PI3K/AKT in HCC Huh7 cells. (a) Agarose gel electrophoresis and RT-PCR analysis (b) showing enhanced transcriptional regulation of PI3K/AKT signaling in Huh7 cells (>4-fold increased). Western blot (c) and the quantification data (d) showing enhanced protein expression of PI3K and AKT in HC C Huh7cells. The error bar represented in the graph is ±SD, **P < 0.01.

**Figure 2:**
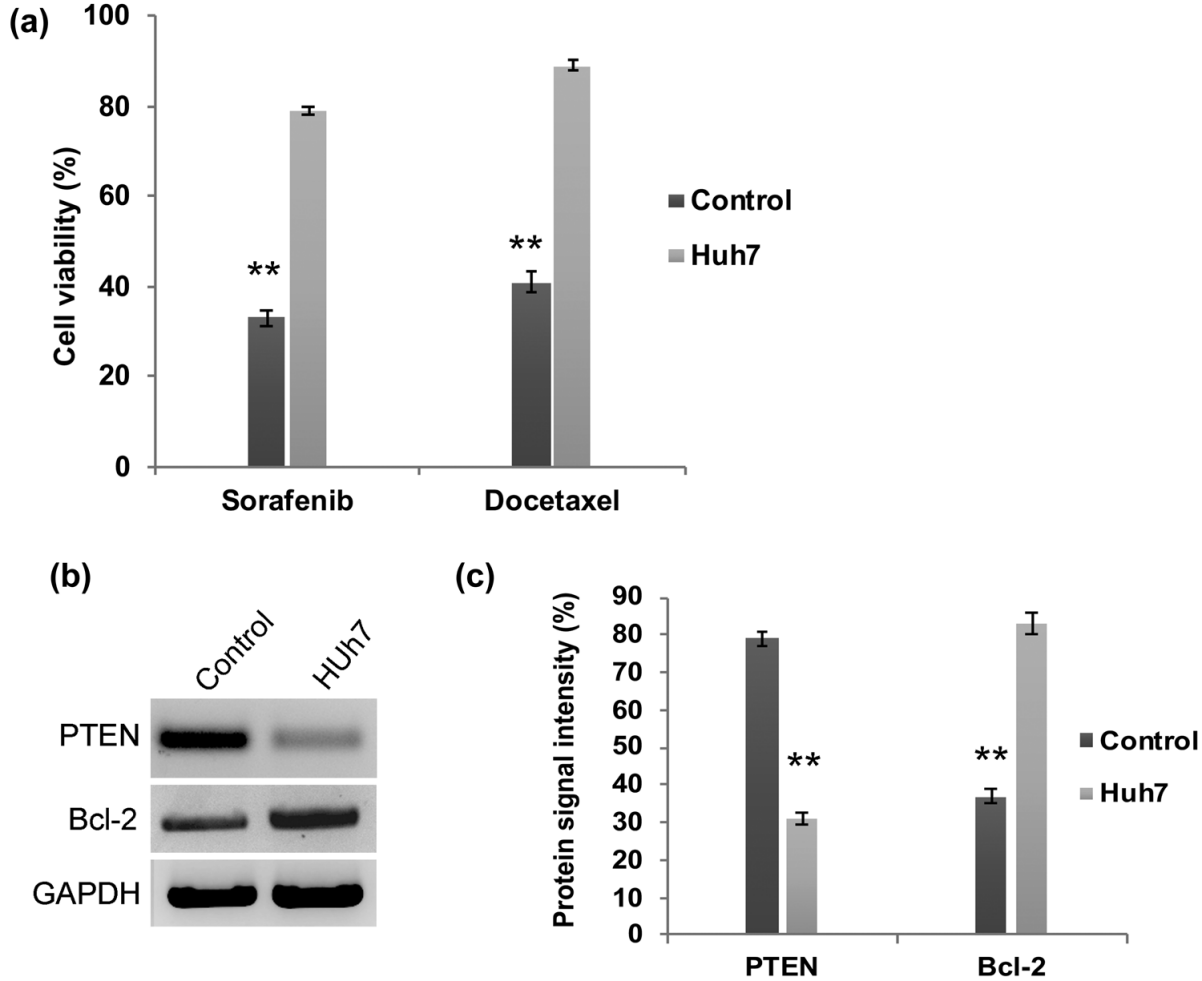
PI3K/AKT activated Huh7 cells are highly resistant to chemotherapy. (a) Quantification graph from chemoresistance assay demonstrating that Huh7 cell viability was not declined upon treatment with docetaxel and sorafenib. Western blot analysis (b) and densitometry bar diagram of blots (c) showing expression of Bcl-2 and PTEN. The error bar represented in the graph is ±SD, **P < 0.01.

We have assessed the transcriptional regulation of PI3K and AKT in HCC Huh7 cells. Our q-PCR analysis revealed that the mRNA expression folds of *PI3K* and *AKT* are significantly (*P* < 0.01) upregulated in Huh7 cells when compared to control cells (Figure 1a and b). Meanwhile, the western blot analysis confirmed the enhanced protein expression of PI3k and Akt in Huh7 cells (Figure 1c and d). Hence, these findings reflect the previous reports that have demonstrated the aberrant activation of the PI3K/AKT signaling pathway in liver cancer cells [15,16,17]. One step further, these PI3K/AKT elevated Huh7 cells were subjected to chemoresistance assay. Upon treatment with docetaxel and sorafenib, the Huh7 cells became more resistant and their cell viability was significantly (*P* < 0.01) higher in Huh7 cells (Figure 2a). Reports in several cancers explained that activated PI3K/AKT signaling is involved in the downregulation of tumor suppressor PTEN and upregulation of anti-apoptotic factor *BCL-2*, respectively. Hence, we have examined the expression pattern of PTEN and Bcl-2 by western blot. PTEN expression is decreased dramatically in Huh7 cells, whereas the anti-apoptotic protein Bcl-2 is highly enhanced (Figure 2b and c). Therefore, these data suggest that aberrant upregulation of PI3K/AKT signaling molecules is involved in HCC apoptosis resistance through the decreased expression of tumor suppressor protein PTEN.

### 3.2 Overexpression of PTEN contributes to improved chemotherapy sensitivity and apoptosis induction in Huh7 cells

**Figure 3:**
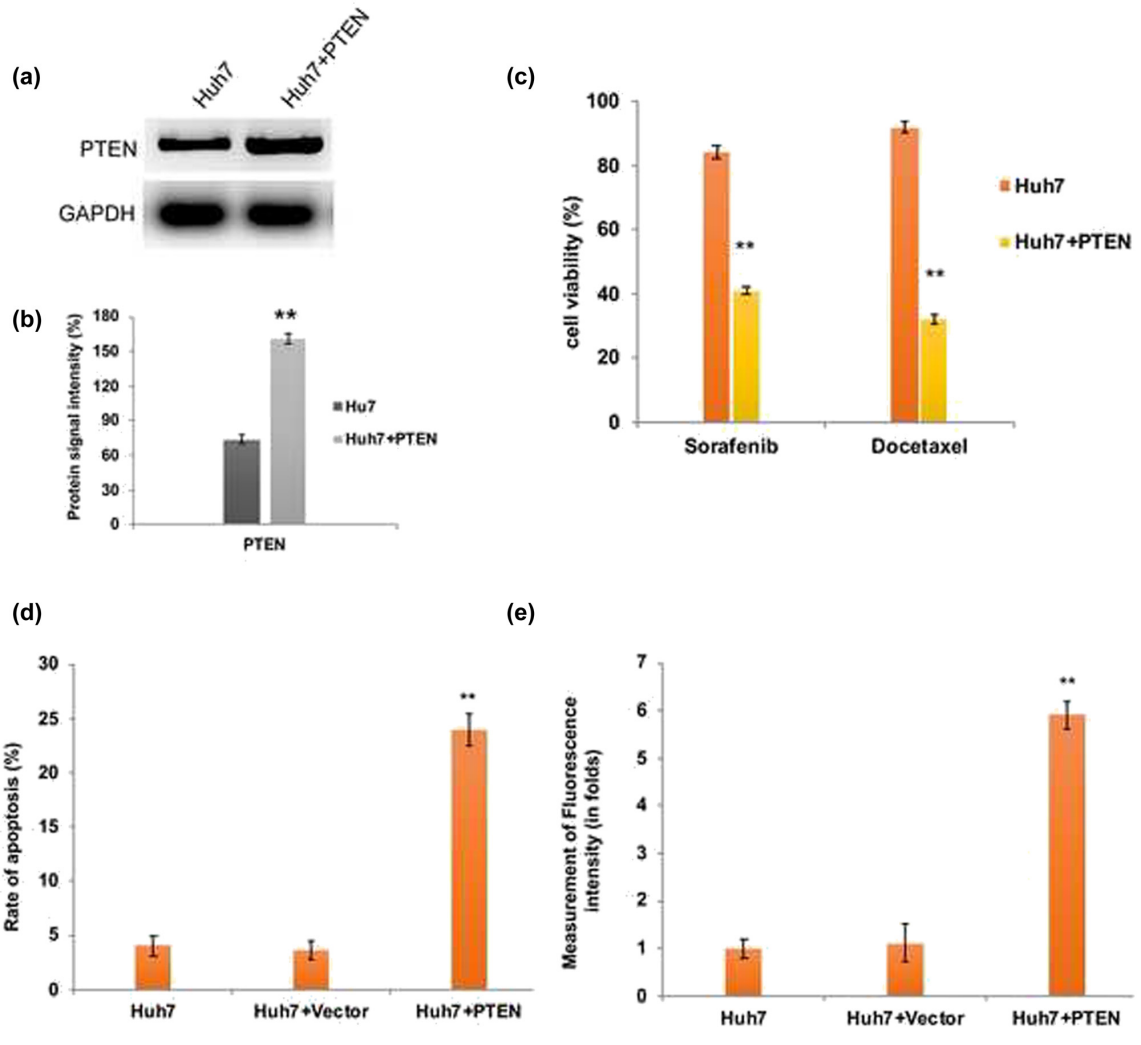
PTEN overexpression induced apoptotic cell death in Huh7 cells. (a) Western blot analysis and densitometry bar diagram of blots (b) showing the overexpression of PTEN in Huh7 cells after transfection with pcDNA3-GFP-PTEN. Quantification graphs from chemoresistance assay (c), assessment of apoptosis by flow cytometry (d), and intracellular ROS measurement by flow cytometry (e) confirm the induction of elevated apoptosis in PTEN overexpressed Huh7 cells due to enhanced ROS generation. The error bar represented in the graph is ±SD, **P < 0.01.

The major inferences of the chemotherapy treatment regimens include apoptosis/multidrug resistance and tumor recurrence when the therapy is withdrawn. From the aforesaid findings, we speculate that apoptosis resistance of Huh7 cells might be due to the downregulation of PTEN. Therefore, we have transfected Huh7 cells with GFP-PTEN overexpression cassette plasmid and examined its effect on chemotherapy resistance and apoptosis induction. As we expected, upon PTEN overexpression (Figure 3a and b) Huh7 cells became more sensitive to docetaxel and sorafenib and the cell viability was significantly (*P* < 0.01) decreased compared to parental Huh7 cells (Figure 3c). In addition, our flow cytometry data revealed that upon PTEN overexpression, the intracellular ROS generated was significantly higher in Huh7 cells (Figure 3d). As a consequence, the rate of apoptosis induction was significantly (*P* < 0.01) elevated in PTEN overexpressed Huh7 cells (Figure 3e).

**Figure 4:**
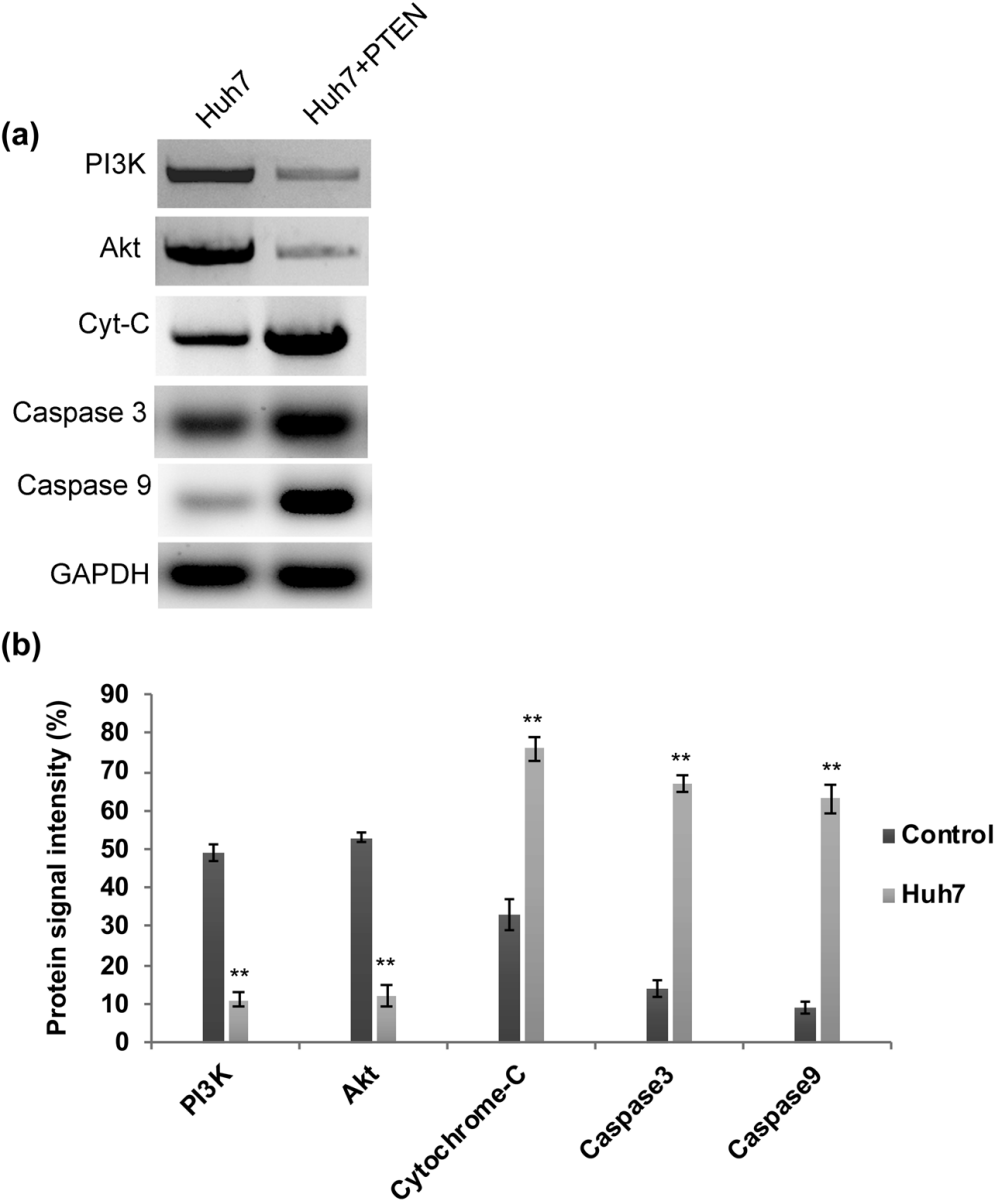
(a) Western blot and its quantification graph. (b) Displaying the apoptosis induction through caspase activation and downregulation of PI3K/AKT signaling.

It has been well documented that stimulating cytochrome C leads to activation and release of downstream caspases essential for mitochondria facilitated intrinsic apoptosis [26,27,28]. Thus we performed a western blot to analyze the expression level of caspases in PTEN overexpressed Huh7 cells. We found that the levels of cytochrome C, caspase 3, and caspase 9 are highly elevated in Huh7 cells overexpressing PTEN (Figure 4). Interestingly, the expression level of PI3K and AKT is comparatively reduced in PTEN overexpressed cells (Figure 4a and b). Hence, PTEN overexpression leads to enhanced chemotherapy sensitivity, intracellular ROS generation, and thus ultimately results in apoptotic cell death through caspase activation and downregulation of PI3K/AKT signaling.

## 4 Discussion

Dysregulation of PI3K/AKT signaling molecules is frequently associated with different types of cancers such as lung, breast, ovarian, prostate, uterine leiomyomata, and liver cancers [17,18,19,29,30,31]. Abnormal PI3K/AKT activation has been reported in liver cancer invasion, metastasis, EMT, sorafenib resistance, and angiogenesis [15,16,17]. Consequently, we also found abnormally activated and enhanced PI3K/AKT signaling in HCC Huh7 cells. Furthermore, these cells were highly resistant to docetaxel and sorafenib, and hence apoptosis was impeded by deregulated PI3K/AKT signaling. On the cell membrane, activation of PI3K leads to PIP3 production, which leads to phosphorylation and activation of Akt [13]. The tumor suppressor gene PTEN negatively regulates Akt through dephosphorylation, and therefore inactivation of *PTEN* causes enhanced expression of growth factors, receptors, and cytokines required for Akt phosphorylation, which are crucial for promoting carcinogenesis [32].

In this study, reduced expression of PTEN was observed in Huh7 cells. At the same time, the activated anti-apoptotic mechanism was evident by the enhanced expression of Bcl-2 in Huh7 cells. These results lead to the hypothesis that depleted PTEN expression might contribute to apoptosis inhibition, and therefore, Huh7 cells are highly resistant to chemotherapy drugs. Corroborating our findings, hyperactivation of Akt and downregulated PTEN expression was found associated with poor prognosis, cancer growth or large fibroid, and tumor recurrence and they have been documented [22,33]. Increasing evidence showed that overexpression of PTEN is involved in cell cycle arrest and stimulates apoptosis by P13K/AKT signaling downregulation in liver cancer, breast cancer, renal carcinoma, and glioma cells [34,35]. In our study, when we complemented PTEN function in Huh7 cells, apoptotic cell death was induced which was evident in the chemoresistance assay. The PTEN overexpressed cells were responding well to docetaxel and sorafenib. As a result, enhanced intracellular ROS was produced and this ultimately leads to apoptotic cell death. Concomitantly, studies on liver cancer reported that a combination of docetaxel with PTEN overexpression became an effective adjuvant therapy [36]. In consistence with previous findings, our findings also demonstrated that apoptosis was persuaded upon PTEN overexpression in cancer cells [37].

The possible molecular mechanism behind the apoptosis induction could be that PTEN directly inhibits P13K signaling and interrupts Akt binding to PI3K by dephosphorylation of PIP3. Several studies reported that PI3K and Akt inhibitors worked efficiently, which sensitizes cancer cells to ionizing radiation and induced cell cycle arrest and thus ultimately results in apoptosis [38,39,40,41]. Our findings revealed the downregulation of pro-survival protein Bcl-2 and release of cytochrome C due to altered mitochondrial membrane potential. Furthermore, reports showed that the released cytochrome C causes activation of caspases 9 which stimulates caspase 3 in association with apoptotic protease activating factor-1 [26,27,28]. As a result, apoptotic cell death happened and cell viability significantly declined in PTEN overexpressed Huh7 cells which was evident from the enhanced level of cytochrome C, caspase 9, and caspase 3.

To conclude, our data suggest that an increased level of tumor suppressor protein PTEN contributes to the downregulation of PI3K/AKT signaling, and therefore, tumorigenesis is checked by the stimulation of apoptotic cell death in liver cancer cells. Considering the fact that there is a cross-talk between signaling pathways (PI3K/AKT/mTOR and Wnt/β-catenin/TGF-β) and the influence of ABC transporter proteins in chemoresistance, further detailed studies are required to target multiple pathways simultaneously to disseminate the underlying molecular mechanism of PI3K/AKT signaling-mediated tumor invasion and metastasis.
